# Interobserver variation in the endoscopic diagnosis of gastroduodenal ulcer scars: implications for clinical management of NSAIDs users

**DOI:** 10.1186/1756-0500-4-409

**Published:** 2011-10-13

**Authors:** Yuji Amano, Goichi Uno, Takafumi Yuki, Mayumi Okada, Yasumasa Tada, Nobuhiko Fukuba, Norihisa Ishimura, Shunji Ishihara, Yoshikazu Kinoshita

**Affiliations:** 1 Division of Endoscopy, Shimane University Hospital, Izumo, Japan; 2 Second Department of Internal Medicine, Shimane University, Faculty of Medicine, Izumo, Japan

**Keywords:** NSAIDs, proton pump inhibitor, gastric ulcer scar, duodenal ulcer scar, interobserver diagnostic agreement

## Abstract

**Background:**

A clinical history of peptic ulcer has been reported to be associated with a high rate of ulcer recurrence in nonsteroidal anti-inflammatory drug (NSAID) users. Therefore, it is a very important issue to precisely know the previous history prior to NSAIDs administration. To clarify the possible difficulty to identify the history, we determined the sensitivity and diagnostic concordance of endoscopy for the identification of ulcer scars indicative of previous clinical history of peptic ulcer diseases.

**Methods:**

The first study enrolled 200 consecutive patients with a clinical history of gastric or duodenal ulcers previously confirmed by esophagogastroduodenoscopy. The sensitivity of endoscopy for identifying scars was determined for these patients. In the second study, the extent of interobserver agreement was determined for 47 endoscopists who identified ulcer scars in endoscopic photographs of 30 sites of previous active gastric ulcers and 30 sites of previous active duodenal ulcers. The kappa coefficient of reliability was calculated to measure the interobserver agreement on the diagnosis of ulcer scars.

**Results:**

Out of 190 patients eligible for analysis, 104 (54.7%) were found to have gastric or duodenal ulcer scars on endoscopy; there were no gastric or duodenal ulcer scars seen in the remaining patients (45%). In the second study, the kappa values for endoscopic diagnosis of gastric and duodenal ulcer scars were 0.14 (95% CI 0.13-0.16) and 0.29 (95% CI 0.27-0.32), respectively. The addition of indigo-carmine chromoendoscopy did not provide a statistically significant improvement in diagnostic concordance in patients with gastric ulcer scar since the kappa value for chromoendoscopic diagnosis was 0.15; 95% CI 0.13-0.17 as low as for un-contrasted scars.

**Conclusions:**

The sensitivity and concordance of endoscopic diagnosis of gastric and duodenal ulcer scars are not satisfactory for the use of endoscopy only to identify previous ulcer disease. To avoid the overlooking the previous clinical history of peptic ulcer diseases, the diagnosis of peptic ulcer scar has to be carefully done prior to NSAIDs administration.

## Background

Nonsteroidal anti-inflammatory drugs (NSAIDs) are widely prescribed and have become increasingly associated with adverse gastroduodenal events [1,2]. Gastric and duodenal ulcers have been reported to be found in as high as 17% of chronic NSAIDs users and 12% of low-dose aspirin (LDA) users. Coadministration of proton pump inhibitors (PPIs) reduces the proportion of gastric and duodenal complications associated with NSAIDs and LDA [3,4]. It has been recommended that PPIs should be coadministered with NSAIDs and LDA in patients with clinical characteristics associated with high risk for ulcer development, including old age, past history of peptic ulcer or complications, and concomitant use of aspirin, anticoagulants, and/or antiplatelet agents [5-11]. A past history of peptic ulcer is reported to be the factor most strongly associated with ulcer development [12,13]. Therefore, NSAIDs or LDA users with a past history of gastric or duodenal ulcers should be carefully managed with appropriate coadministration of PPIs [14,15].

Whether or not endoscopic examination is sensitive enough to detect a previous gastric or duodenal ulcer has not been thoroughly investigated. The aims of this study were to determine the sensitivity of endoscopy used as a tool for detecting ulcer scars and to determine interobserver agreement in the endoscopic diagnosis of ulcer scars.

## Methods

### Sensitivity of endoscopic study for detecting ulcer scars

From August 2010 to December 2010, 200 consecutive patients with a past clinical history of gastric or duodenal ulcer confirmed by esophagogastroduodenoscopy at least 12 months previously were enrolled at Shimane University Hospital. Endoscopists, who were blinded to the past histories of the patients, attempted to locate ulcer scars at sites where active open ulcers had been previously detected by endoscopy. Three well experienced endoscopists (YA, GU, and TY) participated in this study and evaluated endoscopic photographs separately. When their diagnosis did not fit completely, they discussed and made the final consensus diagnosis. Patients who had undergone endoscopic surgery for gastroduodenal lesions, gastrectomy, or esophagectomy were not enrolled.

### Interobserver agreement on endoscopic diagnosis

**Figure 1:**
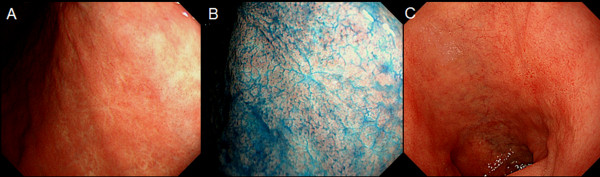
**Representative endoscopic images of the stomach where the active stage of peptic ulcer was found 12 months previously with white light (A) and with chromoendoscopy using indigo-carmine dye (B)**. Representative endoscopic image of the duodenal cap where the active stage of peptic ulcer was found 12 months previously (C).

Forty-seven endoscopists participated in this study. The median length of time they had worked as endoscopists was 9.5 years. They were divided into groups based on duration of experience and the presence or absence of board certification from the Japan Gastroenterological Endoscopy Society (JGES). Endoscopists can obtain JGES board certification after completing 5 years of training in endoscopy at a JGES-approved educational institution and passing an examination administered by the JGES. In the present study, "experienced" endoscopists were defined as endoscopists who had been in practice for over 10 years. "Expert" endoscopists were defined as those with a JGES board certification. Twenty-two out of 47 were experienced and 29 were expert endoscopists. The participating endoscopists diagnosed still endoscopic photographs of 30 cases of previous gastric ulcers and 30 cases of previous duodenal ulcers for the presence or absence of gastric or duodenal ulcer scars. All photographs showed the same gastroduodenal sites where active peptic ulcers had been previously found (Figure 1). In all the gastric ulcer cases, the sites of previous ulcers were photographed with and without indigo carmine staining, and each photograph was diagnosed separately. There were 3 types of diagnosis: confident diagnosis of ulcer scar, confident diagnosis of ulcer scar absence, and equivocal.

### Statistical analysis of interobserver agreement

The kappa coefficient of reliability was calculated to measure interobserver agreement on the diagnosis of ulcer scars. The assessments of a large number of observers with multiple objectives were analyzed using the method proposed by Siegel and Castellan [16], as described in previous reports [17,18]. Kappa coefficients of reliability were determined plus 95% confidence intervals (CIs). Complete disagreement between observers would have a kappa value of -1 and perfect agreement a kappa value of +1. A 0 value would mean agreement by chance alone. A kappa value greater than 0.4 is generally considered to be acceptable for 2-observer analysis. However, the kappa coefficient of reliability tends to be lower in analyses of a large number of observers. Kappa coefficients of reliability were compared between groups, and statistical differences of kappa values between groups were estimated.

The protocol of this study was prepared according to the Declaration of Helsinki and the questionnaires approved by the ethics committee of Shimane University School of Medicine were used. Written informed consent for the endoscopic study was obtained from all participants.

## Results

### Sensitivity of endoscopic study to detect ulcer scars

Out of the 200 patients with a past history of endoscopically confirmed gastroduodenal ulcers, 10 patients were found to have recurrent gastric and/or duodenal ulcers and were excluded from analysis. Out of the remaining 190 patients, 104 were found to have ulcer scars on endoscopy. Ulcer scars were not identified in the remaining 86 cases, resulting in a sensitivity of 54.7% for the endoscopic diagnosis of the previous presence of ulcers, when the examination focused on identifying ulcer scars.

### Interobserver agreement on endoscopic diagnosis

**Figure 2:**
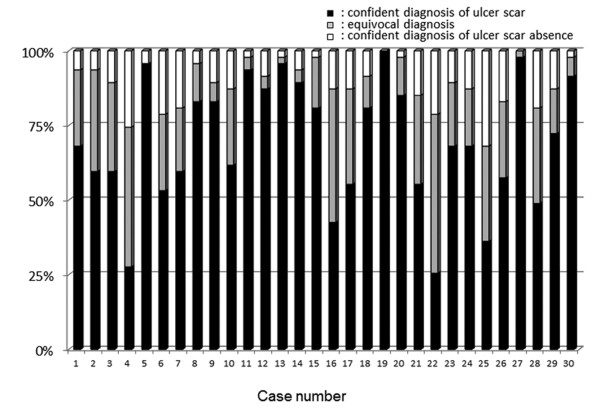
**Endoscopic diagnostic concordance regarding presence or absence of gastric ulcer scar in 30 cases**. (black box): confident diagnosis of ulcer scar, (gray box): equivocal, (white box): confident diagnosis of ulcer scar absence.

**Figure 3:**
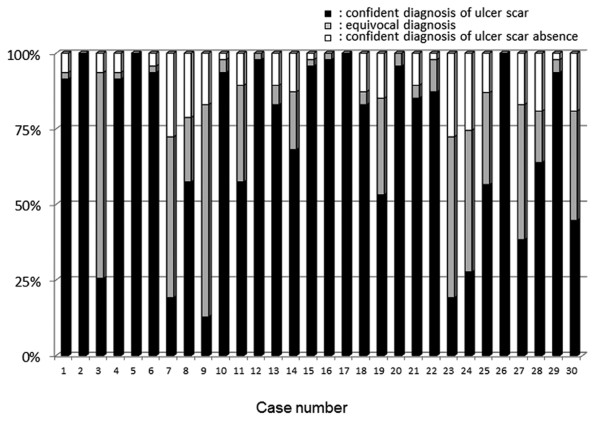
**Endoscopic diagnostic concordance regarding presence or absence of duodenal ulcer scar in 30 cases**. (black box): confident diagnosis of ulcer scar, (gray box): equivocal, (white box): confident diagnosis of ulcer scar absence.

**Figure 4:**
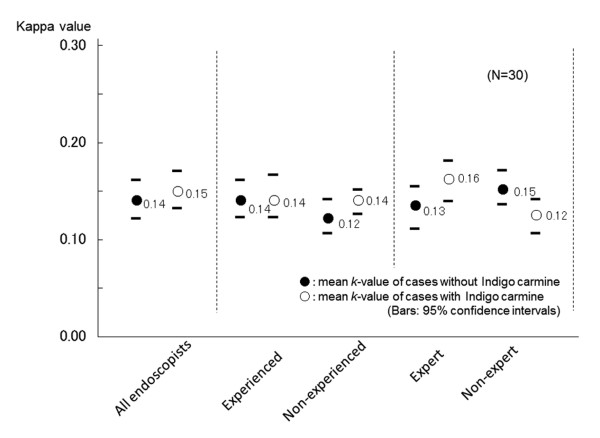
**Kappa values for the endoscopic diagnosis of gastric ulcer scars by white light endoscopy (black circle) and by chromoendoscopy using indigo carmine dye (white circle)**. Bars depict 95% confidence intervals.

A confident endoscopic diagnosis of ulcer scars was possible in 70% (95% CI 61.5%-77.2%) and 72% (95% CI 60.2%-81.9%) of patients with previous gastric and duodenal ulcers, respectively (Figure 2 and 3). The kappa value of the endoscopic diagnosis on the presence or absence of a gastric ulcer scar was 0.14 (95% CI 0.13-0.16) in all the participating endoscopists (Figure 4). In the experienced and the expert endoscopists, the kappa values were 0.14 (95% CI 0.12-0.16) and 0.13 (95% CI 0.11-0.16), respectively. There were no significant differences found between experienced and inexperienced, or expert and inexpert endoscopists. When endoscopists diagnosed using indigo carmine chromoendoscopic images of the same area, the kappa value of all participating endoscopists was 0.15 (95% CI 0.13-0.17), and there was no statistically significant difference compared to that without chromoendoscopy. Moreover, the kappa values with chromoendoscopy of the experienced and expert endoscopists were 0.14 (95% CI 0.12-0.16) and 0.16 (95% CI 0.13-0.18), respectively. They did not show statistically significant differences between those of the inexperienced: 0.14 (95% CI 0.12-0.15) and inexpert endoscopists: 0.12 (95% CI 0.14-0.10).

**Figure 5:**
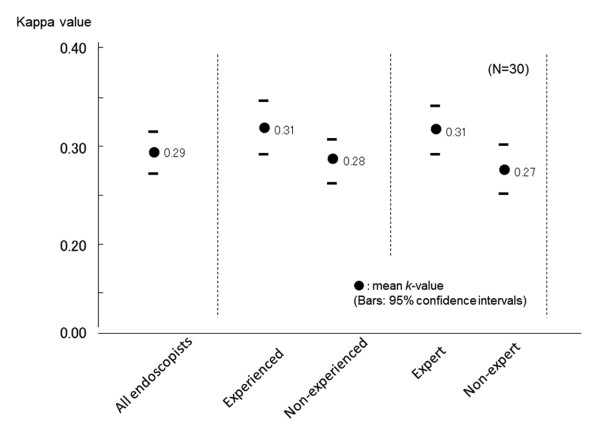
**Kappa values for the endoscopic diagnosis of duodenal ulcer scars by white light endoscopy**. Bars depict 95% confidence intervals.

Interobserver agreements on the endoscopic diagnosis of duodenal ulcer scars are shown in Figure 5. The kappa value for all the participating endoscopists on the diagnosis of duodenal ulcer scars was 0.29 (95% CI 0.27-0.32), which was significantly higher than the value of gastric ulcers as shown in Figure 4. Experienced and expert endoscopists showed higher kappa values, 0.31 (95% CI 0.29-0.35) and 0.31 (95% CI 0.29-0.35), than inexperienced and inexpert endoscopists, 0.28 (95% CI 0.26-0.31) and 0.27 (95% CI 0.25-0.30), respectively, although statistically significant differences were found.

## Discussion

The confident endoscopic diagnosis of ulcer scars was only possible in 54.7% of patients with previous gastroduodenal peptic ulcers, and the kappa value of the endoscopic diagnosis on the presence or absence of gastroduodenal peptic ulcer scar was too low. Thus, in this study, we demonstrated that endoscopic examination for the detection of gastric and duodenal ulcer scars was neither a sensitive nor reliable enough diagnostic modality for identifying a past history of peptic ulcers.

Recurrence of peptic ulcers caused by *Helicobacter pylori *infection can be effectively prevented by *Helicobacter pylori *eradication therapy. Therefore, chronic administration of NSAIDs and LDA are now the important factors associated with ulcer recurrence [5-7]. Patients with a past history of peptic ulcers are considered to have the highest risk for ulcer recurrence and complications when exposed to chronic NSAIDs or LDA administration [8,10]. Therefore, prophylactic administration of PPIs is recommended for chronic NSAIDs or LDA users who have a previous history of peptic ulcer. Diagnosing a past history of ulcer disease can be accomplished by 2 different methods. First, obtaining an accurate patient history of gastroduodenal ulcer disease is considered to be a good approach. However, in daily practice, not all patients with a previous gastric or duodenal ulcer can be accurately identified as having a history of ulcer disease. Abdominal symptoms do not occur in every patient with an ulcer, and as high as 40% of patients have been reported to have no abdominal complaints, even in the presence of endoscopically identified active ulcers [19]. Therefore, obtaining a medical history from the patient is not sensitive enough for appropriate prophylaxis of NSAIDs- and LDA-related ulcer recurrence.

A second possible method for the diagnosis of previous peptic ulcer disease is endoscopic identification of ulcer scars. Ulcer scars found in the stomach and duodenum are characterized by whitish or reddish color changes, smooth converging folds, deformity of the gastric or duodenal wall, presence of regenerating epithelium, and presence of a linear depression [20-22]. Theoretically, therefore, the endoscopic identification of a gastroduodenal scar should not be difficult. In this study, we examined whether the diagnosis of previous ulcer disease by endoscopic identification of ulcer scars was easy and feasible. Contrary to our initial assumption, the diagnostic sensitivity of endoscopy for ulcer scars was only 55%, and diagnostic concordance among endoscopists was far below the acceptable level even if the chromoendoscopy with indigo carmine was added. We previously demonstrated a low diagnostic concordance among endoscopists using endoscopy to identify low-grade reflux esophagitis and short-segment Barrett's esophagus [17,18]. In evaluating endoscopy as a diagnostic modality for previous ulcer disease, we again have discovered problems in the diagnostic reliability of ulcer scars. Since neither endoscopy nor obtaining a medical history from the patient are sensitive enough by themselves to discover previous ulcer disease, at present we must use both methods to compensate each other, especially in patients who need chronic NSAIDs or LDA therapy.

There are limitations to this study. The endoscopic images were still photographs, but not dynamic observation. Therefore, the results of the present study need to be confirmed by video endoscopy. JSGE and other medical institutions have rarely offered training in the endoscopic identification of ulcer scars. Proper training and education may improve diagnostic sensitivity and concordance, and its effects should be evaluated in future.

## Conclusions

We have demonstrated that the sensitivity and concordance of endoscopic diagnosis of gastric and duodenal ulcer scars are not satisfactory for the use of endoscopy as a sole diagnostic modality for previous ulcer disease. To avoid the overlooking the previous clinical history of peptic ulcer diseases, the diagnosis of peptic ulcer scar has to be carefully done prior to NSAIDs administration.

## List of abbreviations

NSAID: nonsteroidal anti-inflammatory drug; LDA: low-dose aspirin; PPI: proton pump inhibitor.

## Conflict of interests

The authors declare that they have no competing interests.

## Authors' contributions

YA and YK designed the research; GU, TY, MO, NF, and NI collected cases and examined endoscopic study; YA and SI analyzed the diagnostic variance; YA wrote the first draft of this paper; YA and YK drafted and revised the manuscript and approved the final version. All authors read and approved the final manuscript
